# Synthesis and Ceramic Conversion of a New Organodecaborane Preceramic Polymer with High-Ceramic-Yield

**DOI:** 10.3390/molecules23102461

**Published:** 2018-09-26

**Authors:** Jing Li, Ke Cao, Jie Li, Meifang Liu, Shuai Zhang, Junxiao Yang, Zhanwen Zhang, Bo Li

**Affiliations:** 1 Research Center of Laser Fusion, China Academy of Engineering Physics, Mianyang 621900, Sichuan, China; jingli0218@163.com (J.L.); ljzsc0102@163.com (J.L.); liumeifang@caep.cn (M.L.); zhangshuai_scu@126.com (S.Z.); bjzzw1973@163.com (Z.Z.); 2 State Key Laboratory of Environment-friendly Energy Materials & School of Material Science and Engineering, Southwest University of Science and Technology, 59 Qinglong Road, Mianyang 621010, Sichuan, China; caoke@swust.edu.cn

**Keywords:** boron carbide, organodecaborane preceramic, high-ceramic-yield, ceramic conversion

## Abstract

Boron carbide is one of the hardest materials known, with diamond-like mechanical properties and excellent chemical stability. It is wildly used in military defense area, nuclear industry, aerospace technology, etc. Precursor-derived ceramics have made it easier to produce pure boron carbide in processed forms and expand its applications. The challenge of this method is the synthesis of precursor polymer with high-ceramic-yield. The aim of the present work is to develop a new poly(6-norbornenyldecaborane-*co*-decaborane) [P(ND-*co*-D)] copolymer, which was successfully synthesized via ring-opening metathesis polymerization of 6-norbornenyldecaborane and tandem hydroboration with decaborane. The obtained light-yellow powder displayed good solubility, and was fully characterized by NMR, FT-IR and GPC analysis. Thermogravimetric analysis demonstrated that the char yield was up to 79%. The polymer-to-ceramic transformation process and pyrolysis mechanism has shown that the rearrangement of carbon chains of P(ND-*co*-D) mainly occurred in the temperature range of 350 °C~470 °C. Furthermore, the crystallization behavior and microstructures of derived ceramics were studied by XRD and SEM. Nano-sized boron carbide powders were prepared by pyrolysis of P(ND-*co*-D) under argon at 1400 °C for 2 h, while the structure and morphologies of the obtained rhombohedral B_4_C were investigated.

## 1. Introduction

Boron carbide (B_4_C) is an important non-oxide ceramic with excellent thermal-chemical stability, extraordinary mechanical strength, low density and a high cross-section for neutron absorption. Many efforts have been invested to develop this material in armor, thermoelectric, semiconducting, biological and radiation-resistant material applications [1,2]. Commercial B_4_C powder is usually produced by one of the following methods: solid-state reaction from elements, carbothermic reduction, magnesiothermic reduction, various versions of gas-phase interaction, and co-reduction through autoclave synthesis [3,4]. All of these methods need a long duration heat treatment at relatively high temperature, which might bring in impurities like oxygen or metal to the obtained powders. Besides, the formation of boron carbide ceramics with complex shapes, such as films and fibers, is more difficult.

In 2000, Sneddon and coworkers [5] reported a simple and straightforward method for generation of aligned, monodispersed boron carbide nanofibers based on the use of a new single-source molecular precursor. During the past decades, the design of new precursor systems suitable for synthesis of high performance non-oxide ceramics has been a focus of considerable attention [6,7,8]. Recently, Costakis Jr. et al. [9] reported on the formation of near-net shape boron carbide specimen by direct ink writing via suspensions of B_4_C solid and polyethlenimine. Chen et al. [10] used an acrylamide-B_4_C system to produce hollow boron carbide microspheres. Wang et al. [11] prepared porous boron carbide ceramics by a linear organodecaborane block copolymer. It can be seen that the polymer-derived ceramic (PDC) route [12] is one of the most significant and feasible methods to prepare B_4_C materials with complex structures. No matter what the precursor is, one single-source or a mix of B_4_C powders and organic polymers, the important feature of such routes related to the ceramic yield which ultimately determines the utility of the process as well as the bulk properties and shape retention of the resulting ceramics. However, there are few reports on the synthesis and ceramic conversion characteristics of organodecaborane pre-ceramic polymers with high ceramic yields.

In 2014, we synthesized a novel poly(alkenyldecaborane) with decaborane in the mainchain with excellent char yields via platinum catalyzed sequential hydroboration of decaborane, however, the molecular weight was not good and this limited its further application [13]. Inspired by this work, we envisioned that the ruthenium of the Grubbs catalyst might play a dual role for ring-opening metathesis polymerization of alkenyldecaborane and subsequent hydroboration of decaborane with double bonds in the polymer main chain.

In this study, a new copolymer poly(6-norbornenyldecaborane-*co*-decaborane) [P(ND-*co*-D)] was successfully synthesized via ring-opening metathesis polymerization of 6-norbornenyl-decaborane and tandem hydroboration with decaborane. B_4_C powders have then been directly made by the pyrolysis of this copolymer.

## 2. Results

### 2.1. Characterization of P(ND-co-D) Precursor

**Scheme 1:**
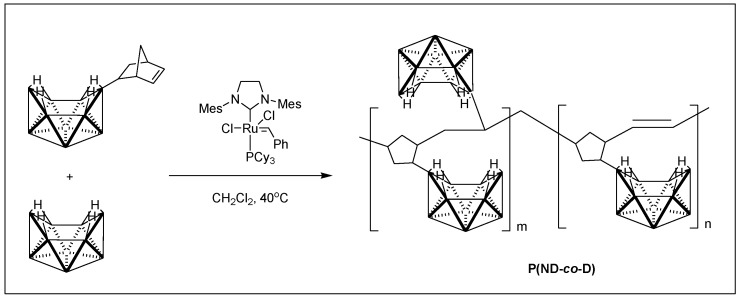
The synthesis of P(ND-*co*-D) precursor.

The synthetic route is illustrated in Scheme 1.

**Figure 1:**
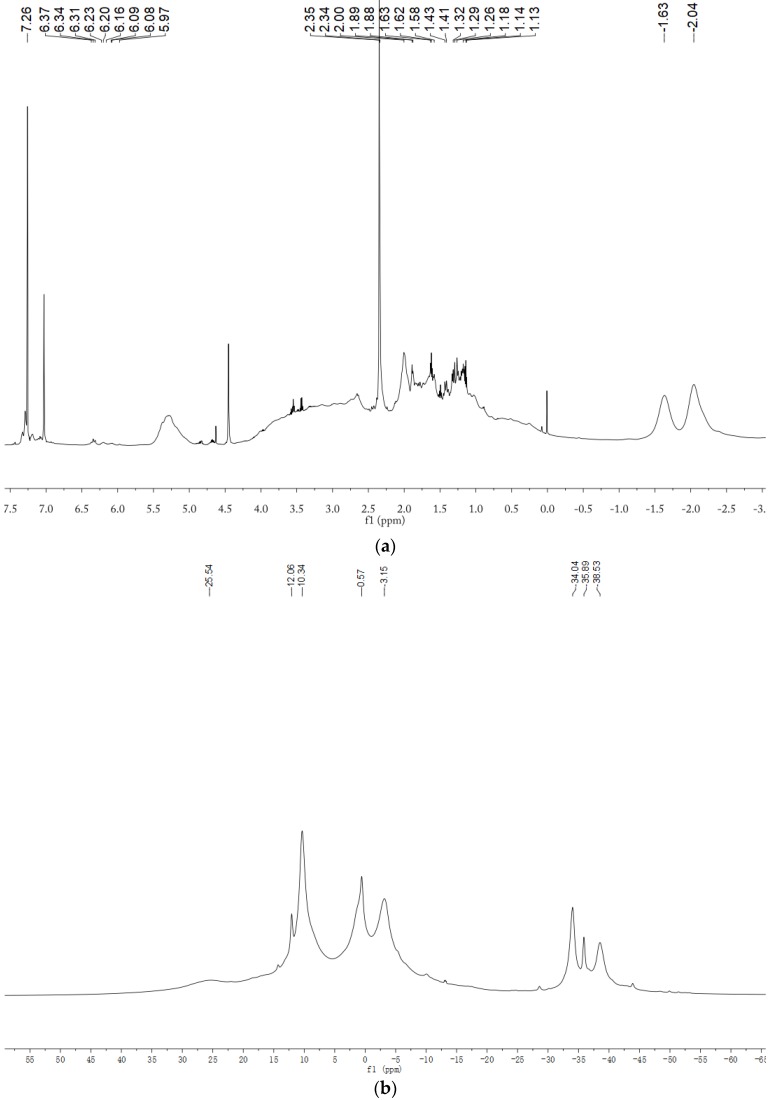
NMR spectra of P(ND-*co*-D) precursor: (**a**) ^1^H-NMR; (**b**) ^11^B{^1^H}-NMR.

Figure 1 shows the ^1^H-NMR and ^11^B{^1^H}-NMR spectra of the P(ND-*co*-D) precursor [14,15]. Comparing to the ^1^H-NMR data of poly(6-norbornenyldecaborane) (PND) [12], we can see that in addition to the characteristic peaks of PND, there are several sets of peaks belonging to disconnected double bonds between 5.8–6.5 ppm, which demonstrate the hydroboration of PND with decaborane really occurs and in a random manner. Meanwhile, the ^11^B{^1^H} NMR chemical shifts are consistent with the data of PND. These results indicate the copolymer product was successfully synthesized.

According to the GPC analysis, the weight-average molecular weight (M_w_) of the P(ND-*co*-D) was 4.9 × 10^4^ with the polydispersity index (PDI) of 2.44. Moreover, P(ND-*co*-D) can be well dissolved in DMF and DMSO at room temperature (0.05 g of P(ND-*co*-D) in 1 mL of solvent).

### 2.2. Thermal Analysis of P(ND-co-D)

**Figure 2:**
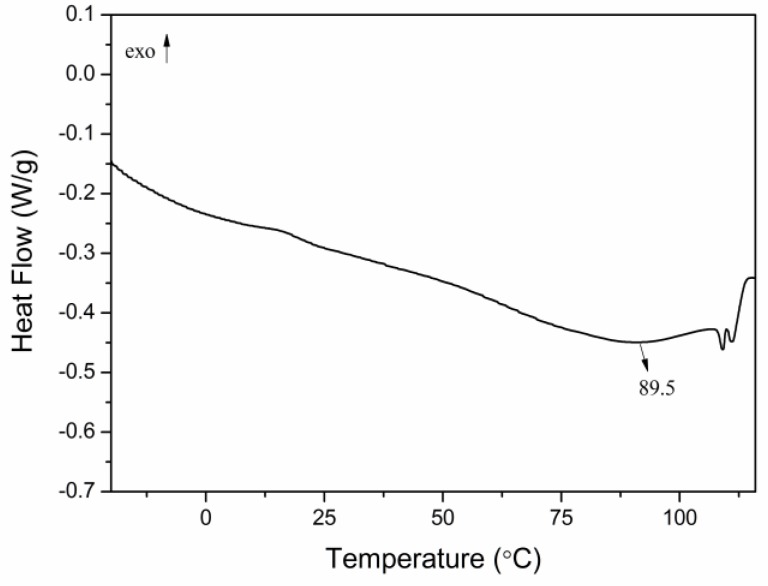
DSC curve of P(ND-*co*-D) precursor.

The DSC curve of P(ND-*co*-D) in Figure 2 shows that there was a glass transition peak at about 89 °C, while the endothermic peak of the melting point was at 109 °C. The two melting peaks might be caused by the different crystalline states of the precursor polymer. The lower T_g_ and T_m_ make this copolymer have a great potential to be further processed into any desired form before thermal conversion.

**Figure 3:**
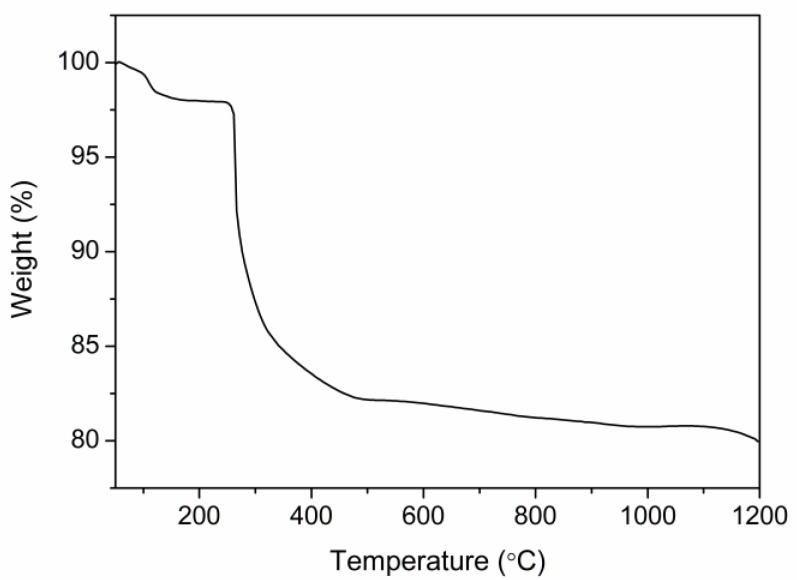
TGA results of the copolymer P(ND-*co*-D) from 50 °C to 1200 °C.

The TGA results of the copolymer P(ND-*co*-D) from 50 °C to 1200 °C is shown in Figure 3. A slight initial weight loss of about 2% was observed in the temperature range below 150 °C, which could be attributed to the decomposition of low molecular weight products. The main sharp decrement between 250 °C and 700 °C referred to the degradation of the precursor, which might be associated with the release of H_2_, small molecular weight alkanes and olefins caused by the breakage of carbon chains [16]. And the char yield of P(ND-*co*-D) is about 79% at 1200 °C, which has distinct enhancement compare to that of poly(6-norbornenyldecaborane) (PND) reported by Sneddon group [14]. This result further supported that the additional decaborane has introduced into the polymer main-chain via ruthenium catalyzed tandem hydroboration. Meanwhile, this relatively high char yield demonstrated that P(ND-*co*-D) displayed excellent thermal properties as well as poly(alkenyldecaborane) with decaborane in the main chain [13]. The extra introduced decaborane improved the crystalline property of the precursor, which is beneficial to the shape retention of polymer-derived boron carbide ceramics.

As we know, boron carbide is normally represented by a B_11_C icosahedral structure and C-B-C intericosahedral chains, with carbon concentrations ranging from 8.8% to 20%. This range of concentrations is made possible by the substitution of boron and carbon atoms for one another within both the icosahedra and the three-atom chains. Because of their high carbon contents, the P(ND-*co*-D) polymers would be expected to produce the more carbon-rich B_4_C composition. Thus, if all of the boron in the precursor polymers was retained and all of the hydrogen and excess carbon was lost during their ceramic conversion reactions, then it would convert to a B_4_C boron-carbide composition with theoretical char yield of 74.7%. However, P(ND-*co*-D) showed a char yield of 79% at 1200 °C which is higher than the predicted one, indicating additional carbon was retained in the ceramic. The high carbon content of these ceramics is consistent with the carbon-rich nature of the P(ND-*co*-D) polymers.

### 2.3. Ceramic Conversion Reactions of the P(ND-co-D)

**Figure 4:**
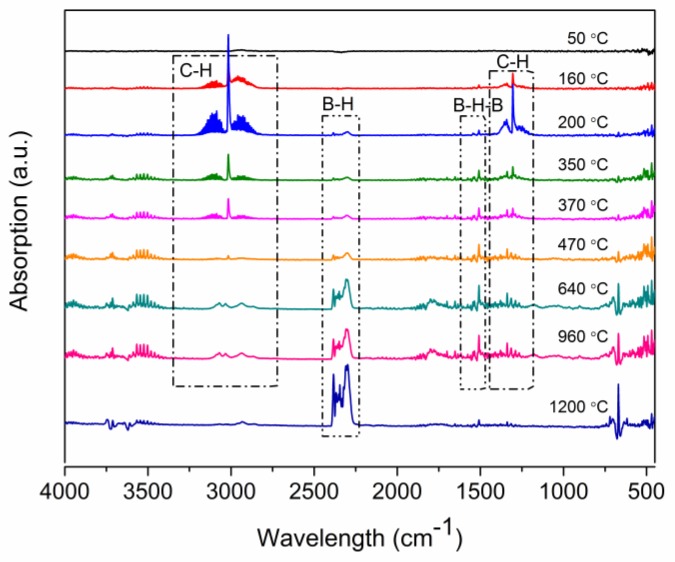
The FTIR spectra of gas products released at different temperatures of pyrolysis.

The FT-IR spectra of pyrolysis gas products at different temperatures are presented in Figure 4. This was used to study the pyrolysis process of the P(ND-*co*-D) precursor at temperatures below 1200 °C. There is almost no gaseous product detected in the initial stage, implying the pyrolysis has not occurred, which is accordance with the TGA results. The stretching vibration peaks of C-H at around 1300 cm^−1^ and 3000 cm^−1^ were attributed to mixture of low molecular weight volatile alkanes (methane, ethane and propane) [17].

The stretching vibration of C-H was enhanced when the temperature reached 200 °C, which indicates the main decomposition process of the precursor occurs. Moreover, the intensity of the absorption peaks decreased in the temperature range of 350 °C~470 °C, which means that a rearrangement of carbon chains in precursor mainly occurred. However, the B-H stretching vibration at 2300 cm^−1^ and B-H-B at 1520 cm^−1^ [16,18] were obviously increased between 640 °C and 1200 °C, which might result from the crosslinking reaction between borane and alkene, and implies that the gaseous products include low molecular weight borane resulted from the decomposition of decaborane cage structures [19].

**Figure 5:**
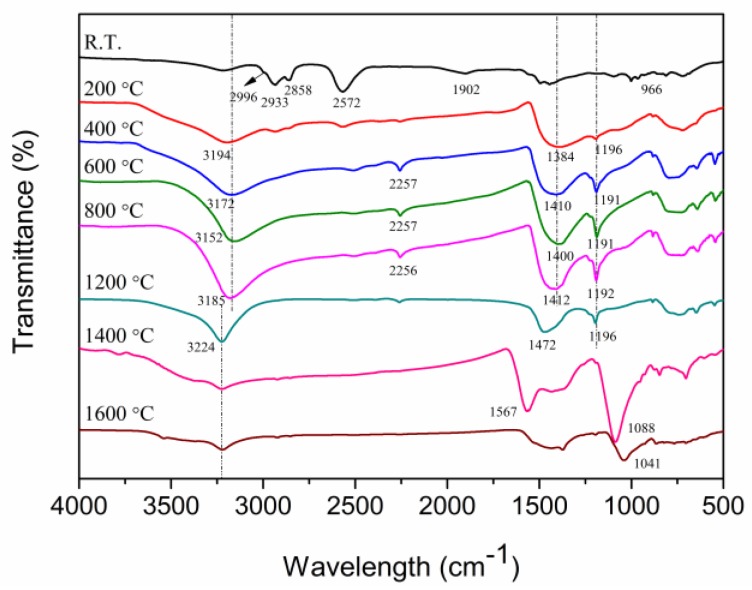
The FT-IR spectra of residues remaining at different pyrolysis temperatures.

The FT-IR spectra of pyrolysis residues at different temperatures are shown in Figure 5. We can see an absorption peak at 2572 cm^−1^ attributable to the stretching vibration of B-H in the decaborane cage [20,21]. Stretching vibration peaks of C-H at 2933 cm^−1^ and 2858 cm^−1^, C=C-H at 2996 cm^−1^ and B-H at 2572 cm^−1^ are weakened at 200 °C. The pyrolyzed sample probably consists of inorganic componnets such as B_4_C and C. The FT-IR spectra of the samples treated at 200 °C~800 °C are almost the same, implying no obvious phase transformation during this process. During this period, double bond crosslinking reactions might be occurring, which would increase the precursor polymer char yield [22]. The broad peak at 1472 cm^−1^, which becomes broader and stronger as the temperature rises, is assigned to the amorphous boron carbide framework structure. It can be seen that the bands assigned to the organic stretching vibration almost disappeared as the sintering temperature increased, and especially at above 1200 °C, the presence of B-C peaks at 1565 cm^−1^ and 1089 cm^−1^ [23] indicate that the ceramization process of P(ND-*co*-D) was complete. When the sample is treated over 1400°C, the broad peak of B-C shifts and gradually becomes weakened. A peak appears at 1041 cm^−1^, indicating the formation of high crystalline B-C [5].

**Figure 6:**
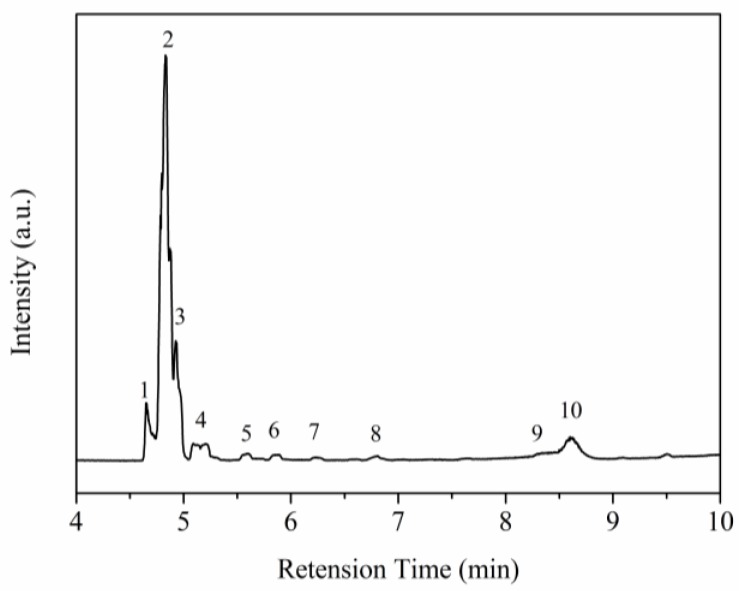
The total ion chromatogram (TIC) of volatile products evolved from TGA during thermal decomposition of P(ND-*co*-D) precursor at 310 °C.

The total ion chromatogram (TIC) of the pyrolysis gas of precursor polymer which corresponds to a TG temperature of 310 °C is exhibited in Figure 6, and the relative compound identification of mass spectrometry according to the PerkinElmer NIST MS library and published data is listed in Table 1.

**Scheme 2:**
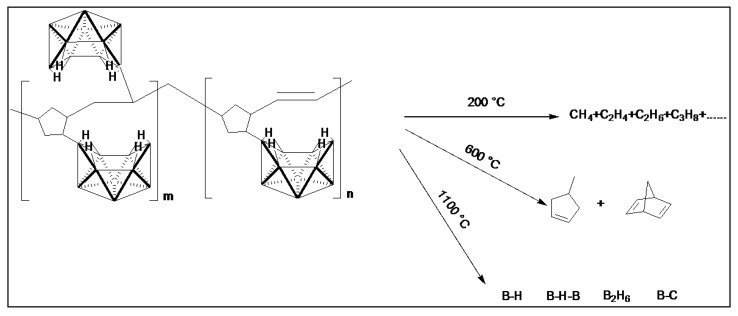
The deducted ceramization process of the P(ND-*co*-D).

It can be seen that the main evolved compounds are low molecular alkanes and olefins with linear carbon chains or ring structures, which means the pyrolytic process just occurred in the backbone of the P(ND-*co*-D) precursor by breakage and rearrangement, while the branched –B_10_H_13_ groups did not participate in the reaction at this temperature. The results match well with the FT-IR spectrum in Figure 4 at the temperature of 200 °C~350 °C. The deducted ceramization process of P(ND-*co*-D) is given in Scheme 2.

**Figure 7:**
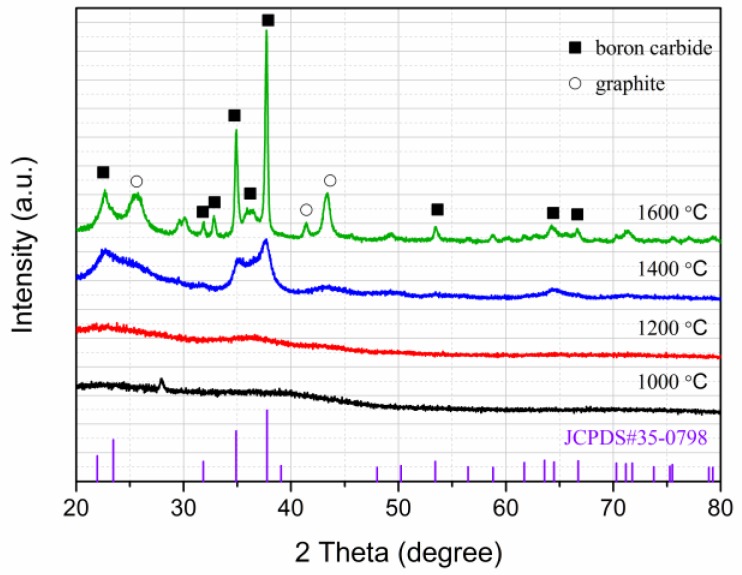
XRD patterns of the P(ND-*co*-D) derived ceramics at different temperatures.

Figure 7 shows the XRD patterns of the precursor powders pyrolyzed at different temperatures from 1000 °C to 1600 °C for 2 h in an argon flow. The precursor powders calcined at 1000 °C and 1200 °C were amorphous.

Peaks corresponding to B_4_C crystal phase (JCPDS No. 35-0798) were recognized with the increasing pyrolysis temperature at 1400 °C, indicating that the formation of rhombohedral B_4_C occurred. The onset of boron-carbide crystallization occurred between 1200 °C and 1400 °C, which is equal to the crystallization temperature of precursor poly(6-norbornenyl- decaborane) (PND) derived ceramics [24]. However, as indicated by various broad diffraction peaks in the XRD patterns, the powder residues obtained at 1400 °C retained a substantial amorphous component. Above 1400 °C, continued refinement of peak shapes and intensities were observed, accompanied by peaks at about 26° and 44° (2θ) assigned to free graphite. Compared with PND [14], although the additional copolymerized decaborane enhanced the ceramic yield of the P(ND-*co*-D) precursor, the crystalline temperature was not highly improved, as well as the processing difficulty was not raised.

**Figure 8:**
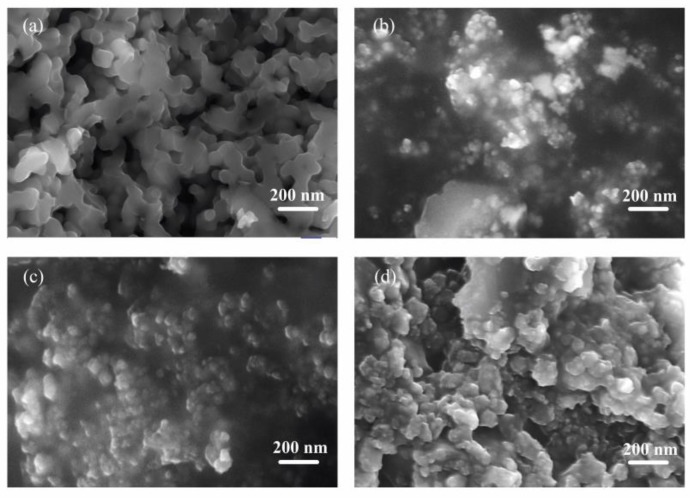
SEM images of ceramic residues at different temperatures derived from the P(ND-*co*-D) precursor polymer: (**a**) 1000 °C; (**b**) 1200 °C; (**c**) 1400 °C; (**d**) 1600 °C.

The SEM micrographs of ceramic residues at different temperatures derived from the precursor polymer (Figure 8) revealed that nanoscale powders were formed. As can be seen in Figure 8, the particles prepared at 1000 °C and 1200 °C appeared amorphous with irregular shape and connected neck. Nanocrystallites embedded in a largely amorphous matrix could be found with the increasing pyrolytic temperature. However, the powders’ size that showed in Figure 8c was much smaller as illustrated in the SEM image of the P(ND-*co*-D) samples pyrolyzed at 1600 °C (Figure 8d), which might infer that the excess carbon in the P(ND-*co*-D) retards the boron-carbide crystallization and the amorphous matrix in Figure 8d was mainly caused by the carbon-rich polymeric precursor. The SEM images are fully consistent with the XRD results.

Composition of the ceramic powders was determine by SEM-EDS analysis (Table 2). Elemental analyses showed that significant amounts of boron increased (from 48.60% to 67.78%) while carbon decreased (from 51.40% to 27.74%) with the increasing pyrolysis temperature. This suggests incomplete ceramic conversion at the lower temperatures with retention of some chemically active species. The lower B:C ratios of the ceramics indicate the high carbon contents of the P(ND-*co*-D) polymer. Powder XRD analyses of the bulk ceramics showed that samples pyrolyzed up to 1400 °C still retained a substantial amorphous component, with the excess carbon in these materials retarding the crystallization process.

## 3. Discussion

A new copolymer P(ND-*co*-D) prepared by ring-opening metathesis polymerization of 6-norbornenyldecaborane and tandem hydroboration with decaborane led to a high-ceramic-yield precursor in 79% yield. Its structure was well confirmed by NMR, FT-IR and GPC. The polymer to ceramic transformation process was investigated by a TG-IR-GC-MS system. We also found that the rearrangement of carbon chains in the precursor mainly occurred in the temperature range of 350 °C~470 °C. During the pyrolytic process, three independent parts, including linear carbon chain, ring structures, boron compounds, could be observed in sequence. The ceramization was completed at 1200 °C, while the crystallization occurred above this temperature. Our results demonstrated that the Grubbs-II catalyst could play a dual role in the ring-opening metathesis polymerization of alkenyldecaborane and subsequent hydroboration of decaborane with double bonds in the main polymer chain. These extra induced decaborane units not only increase the char yield, but also improved the crystallinity of polymer-derived ceramics, which is consistent with some previous research [11,14]. The synthesized polymer P(ND-*co*-D) has proven to be an excellent single-source precursor to boron-carbide ceramic materials such as nanopowders or thin films with high-ceramic-yield.

## 4. Materials and Methods 

### 4.1. Materials

The Grubbs-II catalyst (Sigma-Aldrich, Hannover, Germany), decaborane (Kaimeike Chemical, Zhengzhou, China), 2,5-norbomadiene (Alfa Aesar, Shanghai, China), 1-butyl-3-methylimidazolium tetrafluoroborate (Bmim∙BF_4_, Chengjie Chemical, Shanghai, China), *n*-hexane (Kelong Chemical, Chengdu, China) and ethyl vinyl ether (Aladdin Reagent, Shanghai, China) were used as received without any further purification. Dichloromethane (DCM, Kelong Chemical, Chengdu, China) was dried over calcium hydride. Toluene (Kelong Chemical) was purified by distillation. All synthetic reactions were carried out using standard inert-atmosphere technique as described by Shriver [25].

### 4.2. Synthesis of P(ND-co-D)

#### 4.2.1. Synthesis of 6-Norbornenyldecaborane

According to the method developed by the Sneddon group [6], decaborane (0.12 mol), 2,5-norbomadiene (0.36 mol), toluene (120 mL) and Bmim∙BF_4_ (40 mL) were sequentially added to a 500 mL dried autoclave under an argon atmosphere, then the mixture was reacted at 120 °C for 24 h while being stirred vigorously enough to form an emulsion. When the reaction was complete, the crude product was removed in the toluene layer. After evaporation of the solvent, the residue was purified by column chromatography on 200–300 mesh silica gel with petroleum ether as eluent to give 6-norbornenyldecaborane in 69% yield (18 g).

#### 4.2.2. Synthesis of P(ND-*co*-D)

To a 250 mL dried flask were sequentially added 6-norbornenyldecaborane (20 mmol), decaborane (20 mmol), dichloromethane (150 mL) and Grubbs-II catalyst (5 mol%) under an argon atmosphere. The polymerization reaction was carried out with vigorous stirring at 40 °C for 24 h (Scheme 1), and quenched by adding ethyl vinyl ether (2 mL). After that, the mixture was filtered through a silica gel column with CH_2_Cl_2_ as eluent and after evaporation of the solvent, the residue was precipitated in *n*-hexane. The resulted product was obtained as light-yellow powder in 47% yield (3.5 g).

### 4.3. Preparation of Ceramic

All pyrolysis experiments were carried out at a heating rate of 10 °C/min to the desired temperature between 1000 °C and 1600 °C under high purity argon flow by direct using the synthesized precursor powder. The holding time at the final heating temperature was fixed for 2 h.

### 4.4. Characterization

^1^H-NMR (600 MHz) and ^11^B-NMR (192 MHz) of the synthesized precursor polymers were obtained by nuclear magnetic resonance (NMR) spectrometer (Avance III600 MHz, Bruker, Switzerland). All ^1^H-NMR spectral data are reported in ppm relative to tetramethylsilane (TMS) used as internal standard, and ^11^B{^1^H} NMR spectra data are referenced to external BF_3_∙Et_2_O. Molecular weight and its distribution were determined by gel permeation chromatography (GPC, Model 150 chromatograph, Waters, Milford, MA, USA) with two PL 7.5 mm MIXED-D columns and one 5 mm guard column. Both multi-angle light scattering and a differential refractive index (DRI) detector (Wyatt Technology Corporation, Santa Barbara, CA, USA) were employed with *N*,*N*-dimethylformamide (DMF) as eluent. The spectrum was calibrated with narrow polystyrene standards.

The polymer-to-ceramic transformation studies were performed by thermal gravimetric analysis (TGA, STM 8000, Perkin Elmer, Waltham, MA, USA) with a heating rate of 10 °C/min ranging from room temperature to 900 °C under a helium atmosphere (purity 99.999%, O_2_ ≤ 0.001%), while the gas flow rate was 50 mL/min. Glass-transition temperature (T_g_) of the polymer was measured by a differential scanning calorimetry (DSC, Thermal Analysis Q2000, TA Instruments, New Castle, DE, USA) under a nitrogen flow (50 mL/min) at a heating rate of 10 °C/min from 25 °C to 150 °C. The chemical structure and composition of polymers as well as the pyrolysis residue were analyzed by Fourier transform infrared spectroscopy (FT-IR, Spectrum, Perkin Elmer, Waltham, MA, USA) with a measuring range from 400 to 4000 cm^−1^. A TG-IR-GC-MS system, which combined with TGA (Pyris 1, Perkin Elmer), FT-IR spectrometer (Spectrum 100, PerkinElmer) and gas chromatography-mass spectrometry (GC-MS, Clarus 680, Perkin Elmer), was used to perform the pyrolysis atmosphere products analysis. X-ray diffraction (XRD, Miniflex 600, Rigaku, Tokyo, Japan) patterns were collected with Cuk*α* radiation (λ = 1.54 Å) from 10 to 90° (2θ = 0.02°). The surface morphologies were observed by scanning electronic microscopy (SEM, MerlinVPCompact, Carl ZEISS, Cambridge, UK) with an energy dispersive spectrometer (EDS). The samples were coated with gold.

## Figures and Tables

**Table 1:** Analytical results of the chemical constituents of the pyrolytic products of P(ND-*co*-D) precursor at 310 °C.

No.	RT (min)	Compounds	*m/z*	Formula
1	4.65	Allene	40	C_3_H_4_
2	4.83	Propane	44	C_3_H_8_
3	4.93	Butane	58	C_4_H_10_
4	5.09	Butane, 2-methyl	72	C_5_H_12_
5	5.59	*n*-Hexane	86	C_6_H_14_
6	5.89	Pentane, 2,4-dimethyl	100	C_7_H_16_
7	6.22	Cyclopentane, methyl	84	C_6_H_12_
8	6.81	1,4-Hexadiene	82	C_6_H_10_
9	8.34	Cyclohexane, methyl	98	C_7_H_14_
10	8.60	5-Propyl-1-pentene	112	C_8_H_16_

**Table 2:** The bulk pyrolyses results of the P(ND-*co*-D) copolymer.

Temp (°C)	B% (wt.)	C% (wt.)	B:C	Nominal Composition
1000	48.60	51.40	1.05	B_4_C/C_2.80_
1200	50.82	49.18	1.15	B_4_C/C_2.48_
1400	56.50	43.50	1.44	B_4_C/C_1.77_
1600	67.78	27.74	2.52	B_4_C/C_0.59_
